# Better Prognosis in Newborns with Trisomy 13 Who Received Intensive Treatments: A Retrospective Study of 16 Patients

**DOI:** 10.1007/s12013-012-9355-0

**Published:** 2012-04-10

**Authors:** Keiko Tsukada, George Imataka, Hiroshi Suzumura, Osamu Arisaka

**Affiliations:** Department of Pediatrics, Dokkyo Medical University School of Medicine, Kitakobayashi 880, Mibu, Tochigi 321-0293 Japan

**Keywords:** Trisomy 13, Prognosis, Intensive treatment, Survival, Natural history

## Abstract

Intensive treatment for newborns with trisomy 13 is controversial because of their lethal prognosis. We report the better life prognosis of patients with trisomy 13 who received intensive treatment. At our hospital, we provided an intensive management to such patients including resuscitation and surgical procedures as required. Herein, we present the results of a retrospective study (1989–2010) of 16 trisomy 13 cases who received an intensive treatment. None was diagnosed to have trisomy 13 before birth; 9 were delivered by C-section and oxygen was administered to all patients during postpartum resuscitation. Mechanical ventilation was used in 9 patients after tracheal intubation and tracheotomy was performed in 2 patients when withdrawing of extubation was difficult. Regarding prognosis, 9 patients died, 3 were referred to another hospital, and 4 were discharged from the hospital. Four and 7 patients died within 7 and 30 days after birth, respectively. Nine patients survived for >1 month, 7 for >180 days, and 5 for >3 years. Median survival for 16 patients was 733 days. The patients who received intensive treatments survived longer compared to the previous data. This study provides useful information concerning genetic counseling, especially from an ethical point of view, before providing intensive management to newborns with trisomy 13.

## Introduction

Trisomy 13, first reported by Patau et al*.* [1], is the third common trisomy in live born infants. It is clinically associated with various anomalies, found in 80 %, of cardiac and circulatory systems, such as mainly ventricular septal defect, patent ductus arteriosus, auricular septal defect and 60–80 % with cleft lip and/or palate of mouth, and 50 % or more with the following: central nervous system with holoprosencephaly, hearing system with apparent deafness, cranium with microcephaly with sloping forehead, eyes with microphthalmia and colobomata of iris, retinal dysplasia, abnormal auricle helices, skin with capillary hemangioma of forehead, distal palmar axial triadii, simian crease, hypoconvex narrow fingernails, flexion of fingers with or without overlapping and camptodactyly, polydacyly of hand, skeletal of thin posterior ribs and hypoplasia of pelvis, males with cryptorchidism/abnormal scrotum and females with bicornuate uterus and another malformation accompanied by severe psychomotor disorder [2, 3]. The prevalence studies from Denmark and United Kingdom reported birth prevalence in live born of trisomy 13 as ~1 in 20,000 to 1 in 29,000 [4, 5]. The Hawaiian study reported prevalence at birth of trisomy 13 as 1 in 12,048 in live born infants [6]. Jones described that the incidence was ~1 in 5,000 births [3]. Generally, natural prevalence of delivery at birth of trisomy 13 is between 1 in 5,000 and 1 in 29,000, which is the third most common autosomal trisomy in newborns following trisomy 21 and trisomy 18.

The approach to the management policy of a third-trimester fetus and infant with trisomy 13 and 18 is quite complicated, and the existing literature is controversial. The principal reason for the complexity surrounding decision making in the care of infants with trisomy 13 relates to high neonatal and infant mortality in both conditions [7, 8]. Avoidance of delivery by cesarean section when a fetus is known to have trisomy 13 appears to be the trend in Denmark as shown by obstetrics literature. There was strong consensus among physicians working in perinatal medicine toward abortion for social reasons or because of abnormal prenatal diagnostic results that abortion is acceptable until week 21 in the case of trisomy 13 [9]. Moreover, there tends to be extremely negative information about survival of trisomy 13. It is important to acknowledge that the prognosis is lethal. Approximately, 50 % of infants with trisomy 13 will live longer than a week and 5–10 % of infants will live past 1 year [7, 8]. Tradition, as reflected in the pediatric literature, also indicates a nonintervention approach in the newborn management of trisomy 13. Bos et al. [10] summarized these issues, arguing that early diagnosis was very important so that surgery could be withheld. The important issues that emerge in the counseling and management of newborn infants with trisomy 13 are high infant mortality. Survivors have severe mental retardation, often seizures, and fail to thrive. So that surgical or orthopedic corrective procedures should be withheld in early infancy to await the outcome of the first few months [3].

In Japan, the policies for the treatment of chromosomal anomalies including trisomy 13 and 18 with poor long-term prognoses are determined based on either of two controversial concepts: (i) the provision of thorough love and care while avoiding excessive intensive treatment; or (ii) the provision of active intensive treatment including resuscitation and surgery according to the clinical conditions of an infant and in accordance with the wishes of the infant’s parents [11, 12]. The most important problems in this argument were that there was no evidence about the improvement of prognosis of trisomy 13 treated at the Neonatal Intensive Care Unit (NICU) of Dokkyo Medical University Hospital. In the same context, Kosho et al. [11, 13] investigated the prognosis of intensive treatments in 24 Japanese patients with trisomy 18 at NICU. This report argued better prognosis of trisomy 18 through treatment at NICU based on improved survival period, and the data provide helpful guidelines for clinicians by offering the best information on treatment options for families of patients with trisomy 18. However, there is still a lack of precise data concerning the clinical details and prognosis regarding intensive treatments of patient with trisomy13.

The NICU at the Dokkyo Medical University Hospital provides the intensive treatments including resuscitation of intratracheal intubation, respiratory support, and some kind of surgery for trisomy 13 and 18, if informed consent is obtained from the family after counseling. At NICU, the five tentative treatment policies for trisomy 13 and 18 with poor long-term prognoses of survival are as follows: (1) The treatment policy is determined without relation with trisomy 13 and 18, and is determined after the pathology, treatment options and risks involved have been explained to the parents; (2) The actual initial treatment with resuscitation and medical therapy after delivery are provided to neonates who have ordinary cases, and surgical treatment is also not limited; (3) With respect to surgical treatment and resuscitation, even in case of long-term survival; (4) Psychological care for family is regularly provided by clinical psychotherapists; and (5) Management at home is actively discussed if the family wishes for it.

In this study, in order to determine the survival period of trisomy 13 patients who received intensive treatment, we retrospectively analyzed the detailed clinical data of 16 patients with trisomy 13, admitted to NICU at the Dokkyo Medical University Hospital from 1989 to 2010.

## Patients and Methods

This study comprised 6,230 newborn infants who were hospitalized at the neonatal care unit of Dokkyo University Hospital, Japan during the period of 22 years from 1989 to 2010. Newborn infants presenting with external malformations or organ malformations that were observed during the clinical examinations underwent chromosomal analysis, and 183 patients (2.94 %) were diagnosed with chromosomal anomalies. Of these, 138 patients (2.22 %) were diagnosed with numerical autosomal aberration. A breakdown of these patients of numerical autosomal aberration shows trisomy 21 in 83 patients (1.33 %), trisomy 18 in 39 patients (0.63 %), and trisomy 13 in 16 patients (0.26 %). Other chromosomal abnormalities were 45 patients (0.72 %) including 2 patients with trisomy 8 mosaic and 2 patients with 4p-syndrome and others. In this study, we retrospectively investigated the NICU medical records at our hospital regarding the clinical details of 16 patients with trisomy 13 who received intensive treatment.

## Results

### Family History, Ages of the Parents, History of Pregnancy, and Delivery (Table 1)

None of the 16 patients with trisomy 13 had chromosomal abnormalities in their family histories. The age of the mothers ranged from 20 to 42 years (average: 32.9 years) including mothers who were over 35-years-old for 7 patients and over 40-years-old for 2 patients. The age of the fathers ranged from 22 to 47 years (average: 33.8 years) including fathers who were over 35-years-old for 7 patients and over 40-years-old for 3 patients. Regarding the history of previous delivery, 5 patients had parous mothers. In terms of gender, there were 10 males and 6 females. A definitive prenatal diagnosis of amniotic fluid test for trisomy 13 was not carried out for any of the 16 patients. However, in 15 patients diagnosed with trisomy 13, some type of abnormality was indicated in the ultrasonography test during the course of pregnancy, such as intrauterine growth retardation in 11 patients and brain malformations in 5 patients. The term of delivery ranged from 33 to 40 gestational weeks (average: 35.9 gestational weeks). Regarding the method of delivery, 9 patients were delivered via a cesarean section and 7 patients were delivered via a spontaneous cephalic delivery. The delivery term for the performance of cesarean section ranged from 33 to 40 gestational weeks (average: 35.8 gestational weeks).

**Table 1:** Family history, age of parents, pregnancy, and delivery regarding 16 patients with trisomy 13

Patients	Age of parents (father/mother)	History of previous delivery	Gender	Prenatal ultrasonographic findings	Cesarean section	Apgar score (1 min/5 min)	Gestational age (weeks)	Birth weight (g)
1	31/29	–	M	Hydrocephalus IUGR	–	3/4	35	1,582
2	32/32	–	F	Holoprosencephaly umbilical hernia severe IUGR	+	1/2	35	1,756
3	32/31	–	M	IUGR	+	3/6	40	2,162
4	35/42	+	M	Hydronephrosis	+	1/3	33	3,378
5	41/40	+	M	IUGR oligoamnios	+	2/5	37	2,254
6	22/20	–	F	IUGR	–	6/7	37	2,237
7	33/33	–	F	Hydrocephalus oligoamnios	+	8/9	35	2,540
8	30/35	+	M	–	–	8/9	36	2,380
9	34/32	–	F	Holoprosencephaly IUGR	+	1/3	36	2,784
10	22/21	–	M	IUGR	–	8/8	37	2,342
11	36/35	–	F	Hydrocephalus IUGR polydactyly origoamnios	–	4/9	35	1,960
12	29/33	–	M	Severe IUGR	–	3/7	34	1,602
13	39/37	+	M	Origoamnios				
14	36/36	–	F	Severe IUGR	–	8/10	37	1,746
15	47/34	+	M	IUGR	–	4/8	39	3,366
16	41/37	–	M	IUGR	+	3/6	35	1,950

*M* male, *F* female, *IUGR* intrauterine growth retardation

The average of Apgar Score at the time of birth was 4.25 points for 1 min scores and 6.5 points for 5 min scores. The birth weight ranged from 1,582 to 3,378 g, with an average weight of 2,243 g. The breakdown includes 7 patients weighing 1,500 to 2,000 g, 5 patients weighing 2,000 to 2,500 g, 2 patients weighing 2,500 to 3,000 g, and 2 patients weighting at least 3,000 g.

### Chromosomal Analysis, External Malformations, and Organ Malformations (Table 2)

The chromosomal karyotypes of all 16 patients were diagnosed using a G-band method. The r karyotypes included full trisomy 13 in 14 patients, mosaic type of trisomy 13 in 1 patient, and Robertsonian type of translocation in 1 patient.

**Table 2:** Chromosomal analysis, external malformations, organ malformations with 16 patients of trisomy 13

Patients	Karyotype	(Major external malformations)				(Organ malformations)		
		Head	Face	Abdomen	Extremities others	Brain malformation	Congenital heart disease	Respiratory complications
1	Full trisomy	–	Cleft lip and palate lack of nose	Umbilcal hernia intestinal fistula	Polydactyly	Holoprosencephaly (alober type)	VSD ASD PDA PH	Unseparate superior and inferior with hypoplastic lung
2	Full trisomy	–	Cleft lip and palate	Umbilcal hernia	–	Holoprosencephaly (alober type)	VSD ASD DORV	Respiratory failure
3	Full trisomy	Scalp defect hemangioma	Cleft lip and palate	–	Polydactyly cryptorchidism	–	PDA ECD DORV PS	Tracheal stenosis tracheoesophageal fistula respiratory failure
4	Full trisomy	Scalp defect	–	Prune belly syndrome umbilical hernia	–	–	VSD DORV	Hypoplastic lung tracheal stenosis respiratory failure
5	Full trisomy	Low set ears	Cleft lip and palate	Umbilical hernia	Finger apposition anomaly	–	ECD PS	Respiratory failure
6	Robertson type trisomy	–	–	Necrotizing enterocolitis	–	–	VSD CoA	PPHN respiratory failure
7	Full trisomy	–	Cleft lip and palate	–	–	Holoprosencephaly (alober type)	VSD PDA	Respiratory failure
8	Full trisomy	Scalp defect low set ears	–	Umbilical hernia	Polydactyly narrow fingernails	–	TOF MAPCA	
9	Full trisomy	–	Cleft lip and palate lack of nose	–	–	Holoprosencephaly (semi-lober type)	VSD ASD CoA	Respiratory failure
10	Full trisomy	–	Cleft lip and palate	Inguinal hernia	–	–	TOF	Respiratory failure
11	Full trisomy	–	Cleft lip and palate	–	Polydactyly	–	PDA CoA	PPHN respiratory failure
12	Full trisomy	–	–	Umbilical hernia	–	–	ASD PDA	Vocal cord anomaly
13	Full trisomy	Scalp defect low set ears	Cleft lip and palate	Umbilical hernia	Polydactyly	–	TOF PA	Respiratory failure
14	Full trisomy	–	Cleft lip and palate	–	–	Dandy–Walker malformation	ASD	Respiratory failure
15	Mosaic type trisomy	Scalp defect hemangioma	–	Bowel malrotation inguinal hernia	Narrow fingernails micropenis buried penis	Septum pellucidum fenestration olfactory aplasia	ASD PDA	Respiratory failure
16	Full trisomy	Low set ears	–	Undescended testis	Polydactyly	–	ASD PDA	Respiratory failure

*VSD* ventriculoseptal defect, *ASD* atrial septal defect, *PDA* patent ductus arteriosus, *DORV* double-outlet right ventricle, *ECD* endcardial cusion defect, *PS*: pulmonary stenosis, *CoA* coarctation of the aorta, *TOF* tetralogy of Fallot, *PPHN* persistent pulmonary hypertension of newborn, *MAPCA* major aortopulmonary collateral arteries

The major external craniofacial malformations included scalp defects in 5 patients for the head and cleft lip and palate in 10 patients for the face; malformation of abdomen included umbilical hernia in 8 patients. Polydactyly was noted in 6 patients for the extremities. Among the major organ malformations, 3 patients had alobar and 1 patient had semi-lobar type of holoprosencephaly. Dandy-Walker malformation was observed 1 patient and olfactory aplasia and fenestration of the septum pellucidum in 1 patient for the brain. As for the heart, some type of congenital cardiac disorder was observed in all patients. The breakdown includes atrial septal defect in 7 patients, ventricular septal defect in 6 patients, patent ductus arteriosus in 7 patients, coarctation of the aorta in 3 patients, endocardial cushion defect in 2 patients, tetralogy of Fallot (TOF) in 3 patients, and double-outlet right ventricle (DORV) in 3 patients. Combined respiratory malformations included congenital hypoplastic lung in 2 cases and congenital tracheal stenosis in 2 cases.

### Administration of Oxygen and the Use of Mechanical Ventilation and Surgical Treatment (Table 3)

Signs of acute respiratory failure were observed in 13 patients after birth. To resuscitate, oxygen was administered to all 16 patients. Mechanical ventilators were used for 9 patients to control acute respiratory failure after tracheal intubation. The tracheal intubation was unsuccessful in 1 patient due to severe tracheal stenosis.

**Table 3:** Administration of oxygen and the use of mechanical ventilation and surgical treatment, others with 16 patients of trisomy 13

Patients	Resuscitation of oxygen	Mechanical ventilation	Mode of ventilation	Surgical treatment	Others
1	+	–	–	–	–
2	+	+	IMV	–	Seizures
3	+	–	–	–	–
4	+	+	IMV, HFO	Operation for paracentesis of hydronephrosis	–
5	+	–	–	–	–
6	+	–	–	–	–
7	+	–	–	–	–
8	+	+	IMV	Operation for umbilical hernia	
9	+	+	IMV	–	Seizures
10	+	–	–	–	HOT
11	+	–	–	Plastic operation for cleft lip and palate operation for PDA ligation subcravian method for CoA	–
12	+	+	IMV	–	Seizures
13	+	+	IMV	Blalock–Taussig operation for TOF	–
14	+	+	IMV	–	–
15	+	+	IMV	Tracheotomy operation for inguinal hernia operation for buried penis	Seizures
16	+	+	IMV	Tracheotomy	HOT

*IMV* intermittent mandatory ventilation, *HFO* high frequency oscillation, *HOT* home oxygen therapy, *PDA* patent ductus arteriosus, *CoA* coarctation of the aorta, *TOF* tetralogy of Fallot

The various types of congenital malformations were observed in all 16 patients with trisomy 13; consultations with specialized surgeons were conducted to determine whether surgical treatment would be possible. Due to the difficulty of tube withdrawal after prolonged intubation, tracheotomy was performed for 2 patients. After tracheotomy, respiratory conditions in both patients were stabilized and they were able to survive for long-time period over 7 years. Abdominal operation for umbilical hernia was performed in 1 patient and for inguinal hernia in 1 patient. Plastic operation of the cleft lip and palate was performed in 1 patient in order to improve the cosmetic problem at 210 days of birth before discharge. Cardiac surgery was performed in 2 patients with trisomy 13. Ligation for patent ductus arteriosus and subclavian methods for coarctation of the aorta were carried out for 1 patient but she was died at 592 days of birth because of respiratory failure. Cardiac operation using Blalock–Taussig method was performed for TOF in 1 patient at 330 days of birth and she has survived for more than 3 years and 3 months now.

### Outcomes and Main Cause of Death (Table 4)

In-hospital death at NICU occurred in case of 9 patients, whereas 7 patients were discharged with the condition of homecare. Among them, 3 patients were referred by our hospital to another specialized institution close to the patients’ homes for training purpose of homecare nursing at days 349, 251, and 63 after birth. The ages of 4 patients discharged from our hospital to their homes while they were alive were 336, 331, 256, and 204 days after birth. The average of median hospital stay regarding 7 patients who could be discharged including stay in another specialized institute was 256 days after birth. The survival period of the16 patients with trisomy 13 ranged from death at day 1 (within 24 h of birth) to 10 years and 1 month (this patient lives until now).

**Table 4:** Outcomes and life prognosis with 16 patients of trisomy 13

Patients	Discharge(days)	Transfer another hospital (days)	Home care	Survival time (days)	Main cause of death
1	–	–	–	12 h	Respiratory failure due to hypoplastic lung
2	–	–	–	1	DORV
3	–	–	–	1	DORV tracheal stenosis
4	–	–	–	2	DORV
5	–	–	–	11	Heart failure
6	–	–	–	14	PPHN respiratory failure
7	–	–	–	21	Heart failure respiratory failure
8	–	–	–	39	Heart failure
9	–	–	–	50	Heart failure respiratory failure
10	204	–	+	325	Bacterial infection
11	255	–	+	592 (live)	–
12	63	63	+	1186	Respiratory failure
13	349	349	+	1219 (live)	–
14	251	251	+	1842 (live)	–
15	336	–	+	2,705	s/o central apnea
16	331	–	+	3,713 (live)	–

*DORV* double-outlet right ventricle, *PPHN* persistent pulmonary hypertension of newborn, *s/o* suspect of

The breakdown includes death at 1st day of birth, within 24 h, for 1 patient (6.25 %), death within 7 days of birth for 4 patients (25.00 %), and death within 1 month of birth for 7 patients (43.75 %). On the other hand, 9 patients survived for more than 1 month (56.25 %), 6 patients survived for more than 1 year (37.50 %), and 3 patients survived for more than 5 years (18.75 %). Therefore, the survival rates at ages of 1 day, 1 week, 1 month, 6 months, 1 year, 3 years and 5 years were 93.75, 75.00, 56.25, 43.75, 37.50, 31.25, and 18.75 %, respectively. Median survival time for all 16 patients, both males and females, were 733, 887, and 534 days, respectively.

We also reviewed the data in detail to analyze the main cause of death. Some characteristic trend was recognized as follows: death occurred in 4 patients within 1 week of birth due to a major organ anomaly; especially, 3 patients had DORV and in 2 of 3, intubation with ventilation management was performed. On the other hand, 4 of 6 patients from long-survival group (over 365 days, now living) had some kind of surgery that included tracheotomy in 2 patients and cardiac surgery in other 2 patients. Moreover, 5 of 6 patients from long-survival group (over 365 days) had a history of ventilation therapy.

**Fig. 1:**
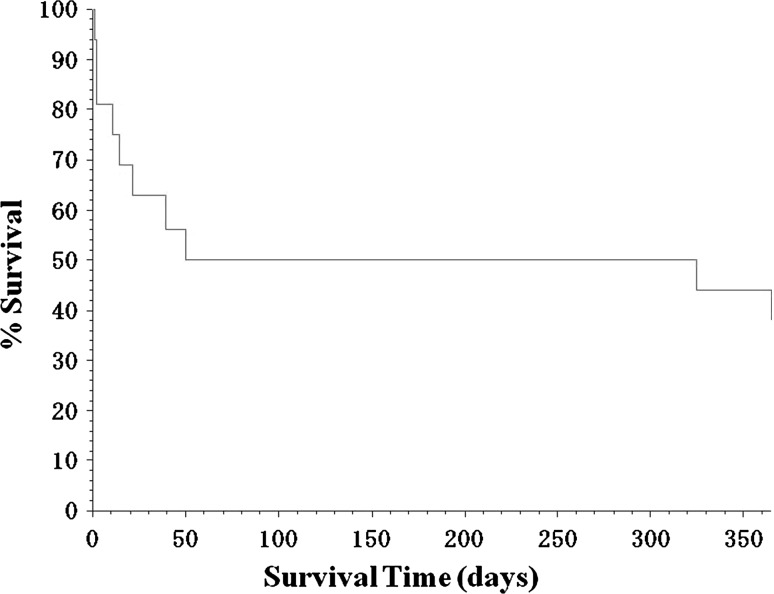
Kaplan–Meier survival curves. Kaplan–Meier survival curves are shown for patients with trisomy 13 who received treatment at the neonatal intensive care unit (NICU) of Dokkyo Medical University Hospital. The data represent 16 trisomy 13 patients. The vertical and horizontal axes represent % survival and survival time (days), respectively, setting the longest survival time at 365 days

**Fig. 2:**
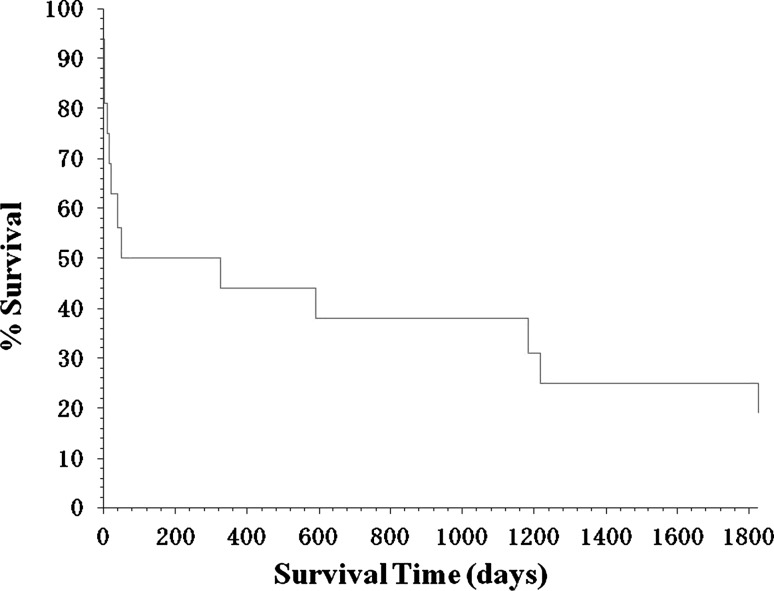
Kaplan–Meier survival curves. Kaplan–Meier survival curves are shown for patients with trisomy 13 who received treatment at the neonatal intensive care unit (NICU) of Dokkyo Medical University Hospital. The data represent 16 trisomy 13 patients. The vertical and horizontal axes represent % survival and survival time (days), respectively, setting the longest survival time at 1,825 days

The longest survival time was set at 365 days (Fig. 1) and 1,825 days equal 5 years (Fig. 2) in the Kaplan–Meier survival curves prepared from the mortality data of all 16 patients with trisomy 13. These Kaplan–Meier survival curves showed mainly two groups: one group that survived for about 2 months and the other group survived for over 365 days.

## Discussion

Among autosomal chromosomal abnormalities, birth is generally believed to be possible in patients of trisomies 21, 18, and 13. Of these, trisomies 18 and 13 are also referred to as fatal chromosomal abnormalities that lead to the development of many severe malformations [14]. Most patients with trisomy 18 and 13 present with severe psychomotor delay, even if they survive for a long period [4, 15, 16]. Historically since the 1980s, resuscitation and surgical treatments for trisomy 18 and 13 have been discussed both from medical and ethical standpoints, taking into consideration the quality of life for the patients and families, medical and economical issues, and the medical standards in different countries. In Japan, the concept of “no treatments beyond current treatments” as proposed by Nishida and Sakamoto [12] of the Tokyo Women’s Medical University has influenced many medical institutions with regard to medical care and management of newborn infants. However, recently, there are few reports of patients with trisomy 13 in Japan who have survived for long periods [17]. Notably, the current problem in Japan is, the primary doctors who tend to patients with trisomy 13 or trisomy 18 provide healthcare by their own medical environments and policies, and no discrete guidelines in this concern have been established. Regarding the medical policy of negative intensive treatment performed without the explanation of detailed medical information, a part of support group members with trisomy 18 or trisomy 13 in Japan have expressed a desire for more active intensive treatment after informed consent.

As for the prognoses of trisomy 13, there is one known report published in 2003 by the American Research Group regarding a large-scale survey of 5,515 cases. According to this report, the average age at the time of death was 10 days, but 5.6 % of the cases survived for more than 1 year [8]. Besides, other long-term survival cases were reported including a 38-year-old adult case of trisomy 13 [18]. There have been several other reports about long-term survivors with trisomy 13 [4, 5, 8, 16, 19]. As summarized in Table 5, our data represent significantly longer median survival periods for the patients with trisomy 13 treated intensively at the NICU.

**Table 5:** Compared survival rates of trisomy 13 in our study with previously reported

Survival time (age)	Denmark [4] *n* = 76 % surviving	England [5] *n* = 16 % surviving	Scotland [16] *n* = 84 % surviving	Atlanta, USA [8] *n* = 114 % surviving	Taiwan [19] *n* = 28 % surviving	Japan (our study) *n* = 16 % survival
1 day	77	69	75	86	89	93.75
7 days	40	38	50	56	61	75
1 month	23	13	28	30	29	56.25
6 months	10	0	ND	11	7	43.75
1 year	ND	0	3	9	4	37.5
3 years	ND	0	ND	ND	4	31.25
5 years	ND	0	ND	ND	ND	18.75
Median survival (days)	2.5	4	8.5	7	9	733

*ND* no data

Of note, two reports have compared between active intensive treatment and non-active treatment with regard to prognosis of trisomy 18 [11, 14] but no such reports are available regarding trisomy 13. Herein, we present for the first time a study of patients with trisomy 13 who received active intensive treatment and discuss the clinical picture and prognosis. Our data suggest that the patients with trisomy 13 who have survived over 60 days after birth may have a high probability of long-term survival. In the previous report on survival of trisomy 18 patients, those who survived longer than 7 months after birth might have a high probability of long-term survival prognosis [14]. In this study, we should not only consider 9/16 (56.25 %) of patients with trisomy 13 who died in the hospital without discharge but also those 7/16 (43.75 %) of patients who were discharged or transferred for purpose of homecare. As our data reveal, one of the major causal factors involved in long-term survival of patients with trisomy 13 is whether the provision of active resuscitation should follow immediately after birth, and then the various surgical treatments should be performed in survivors such as tracheotomy and cardiac surgery. Two previous studies reported intensive cardiac management in patients with trisomy 13 or trisomy 18 [20, 21]. These studies summarized intensive cardiac management including pharmacological intervention, performed to improve the survival periods in patients with trisomy 13 or trisomy 18. In our study, two patients underwent cardiac surgery. One surgical procedure involved ligation of patent ductus arteriosus and subclavian methods for coarctation of the aorta, the other was Blalock–Taussig procedure for TOF; and both survived for long periods: 592 days and more than 3 years and 3 months (living until date), respectively. In addition, all 3 patients with DORV died within 2 days of birth, despite that fact that ventilation management was performed in 2 cases.

In conclusion, although our clinical data represent a small number of trisomy 13 patients, we did observe significantly better survival prognosis in the patients who received intensive treatment management. Our clinical results underscore the need to consider obtaining informed consent and counseling from patients’ families for the future determinations of treatment policy for trisomy 13 patients.
